# 2-Methylisoborneol (2-MIB) Excretion by *Pseudanabaena* *yagii* under Low Temperature

**DOI:** 10.3390/microorganisms9122486

**Published:** 2021-11-30

**Authors:** Ju-Yong Jeong, Sang-Hoon Lee, Mi-Ra Yun, Seung-Eun Oh, Kyong-Hee Lee, Hee-Deung Park

**Affiliations:** 1 Department of Water Environment Research, Gyeonggi Institute of Health and Environment, Suwon 16444, Korea; alfk@gg.go.kr (M.-R.Y.); ose2773@gg.go.kr (S.-E.O.); merculee@gg.go.kr (K.-H.L.); 2 School of Civil, Environmental and Architectural Engineering, Korea University, Seoul 02841, Korea; bfox2@naver.com

**Keywords:** *Pseudanabaena yagii*, 2-MIB odor, 16S-23S rRNA ITS, *mib*C, North Han River, low temperature

## Abstract

Outbreaks of 2-methylisoborneol (2-MIB) contamination in drinking water sources cause inconvenient odor issues in the water distribution system. In this study, microscopy-based isolation with physiological and molecular phylogenetic characterization were performed to investigate and characterize the 2-MIB odor producers that caused an odor problem in the freshwater system of the North Han River in the autumn of 2018. A benthic cyanobacterium was isolated from 2-MIB odor-issue freshwater samples and was found to be phylogenetically affiliated with *Pseudanabaena yagii* (99.66% sequence similarity), which was recorded in South Korea for the first time. The 2-MIB synthesis gene sequences from the odor-issue freshwater samples showed 100% similarity with those in the *P. yagii* strains. Protein sequences of 2-MIB synthase observed in the genome of the isolated strain showed structural and functional characteristics similar to those observed in other *Pseudanabaena* species. The 2-MIB production rate increased slowly during mat formation on the vessel wall; however, it rapidly increased after the temperature dropped. The 2-MIB gene was continuously expressed regardless of the temperature changes. These results suggest that the 2-MIB odor issue in the North Han River might be caused by the release of 2-MIB from the mat-forming *P. yagii* species in a low-temperature freshwater environment.

## 1. Introduction

The presence of earthy-musty odor compounds (geosmin/2-methylisoborneol) in freshwater is a significant problem worldwide, and it has a negative impact on water and aquaculture production [1]. Together with geosmin, 2-methylisoborneol (2-MIB) is known to cause mold odor in freshwater and water distribution systems [2,3,4,5]. Recently, 2-MIB production has been observed in more than 40 species, including benthic and planktonic cyanobacteria [6,7], actinomycetes [8], and fungi [9]. Among them, the cyanobacteria genera are known to be the major 2-MIB producers [10] in freshwater systems. The 2-MIB-producing species are largely non-heterocystous filamentous organisms, and the odor problems from benthic cyanobacteria are fundamentally harder to resolve than those from cyanobacteria of planktonic origin because of their distribution characteristics in the water sample. In the United States, 2-MIB odor problems in water environments are more than twice as likely to be caused by benthic organisms than planktonic organisms [11].

Regardless of whether these substances are toxic, 2-MIB can cause unpleasant odors in tap water without advanced water treatment systems, such as activated carbon [12], UV/H_2_O_2_ treatment systems [13], and biofiltration combined with ozone treatment [14]. However, it has been reported that people with a common sense of smell can detect 2-MIB odor even at low concentrations of several ng/L [15]. Although there is no regulatory standard in South Korea, the recommended standard for 2-MIB odor regulation have been set at 20 ng/L.

Recently, odor issues caused by 2-MIB and geosmin have been reported worldwide because of toxic cyanobacterial blooms due to various environmental changes, such as global climate change and eutrophication [16], and are mostly observed in freshwater environments, such as lakes, reservoirs, and running waters [17]. Especially in South Korea, these phenomena have been mainly observed in lake and reservoir systems. The eutrophication of lakes is caused by the increased residence time of the water body in the artificial lake formed by the dams in the middle of rivers [18]. Eutrophication in lakes leads to abnormal growth of phytoplankton, which in turn causes serious problems in aquatic ecosystems and water resource use [19,20]. Cyanobacteria-like microorganisms are known to be the main producers of 2-MIB and geosmin in lakes and river systems [21], and the relationships between 2-MIB and the occurrence of cyanobacteria have been studied extensively in laboratory-scale experiments [22,23,24]. A previous study reported a relationship between 2-MIB concentration and the number of *Pseudanabaena limnetica* [18]. Nevertheless, research on odor issues in the Korean water system has been less focused on 2-MIB than on geosmin because the 2-MIB odor has rarely been detected in tap water. In addition to statistical significance, there is only a limited amount of molecular biological information about 2-MIB-producing organisms distributed in South Korea [25,26]. However, the microbial populations that are mainly involved in odor compound production are still unclear in natural freshwater systems.

The aim of this study was to reveal the major cause of the earthy-musty odor outbreak in Paldang Lake. Physiological and molecular biological techniques, including qPCR and whole genome sequencing, were applied to investigate 2-MIB odor production in freshwater systems during the autumn season of 2018. These techniques were applied to (1) detect and analyze sequences of 2-MIB synthesis-related genes from North Han River water samples with high 2-MIB odors; (2) identify the odor-producing species by isolating those with the same 2-MIB gene sequences; (3) estimate the 2-MIB production and release characteristics of the isolated odor-causing species; (4) elucidate the mechanism underlying high concentrations of 2-MIB production at low water temperatures in the autumn season; and (5) elucidate the genetic and biochemical background of 2-MIB biosynthesis in the *P. yagii* isolated in this study using whole genome sequencing.

## 2. Materials and Methods

### 2.1. Sampling Sites and Isolation of 2-MIB Producing Cyanobacteria

**Figure 1:**
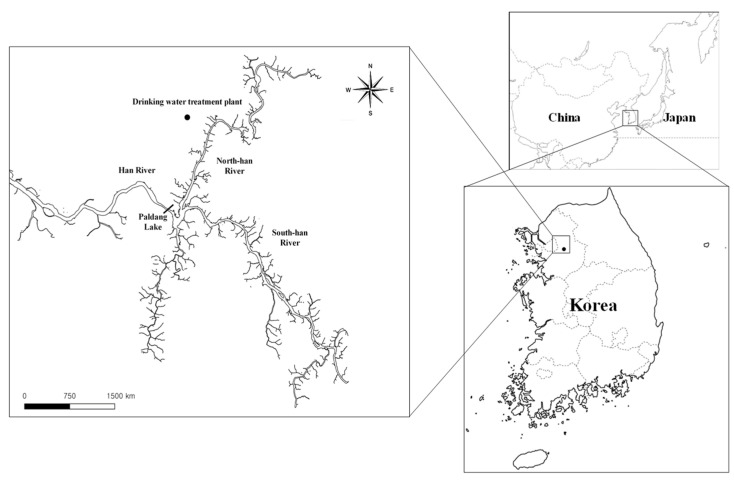
Sampling sites (black circle) in the water stream of the North Han River used in this study.

To isolate the microorganisms that produce 2-MIB and to survey the 2-MIB synthesis gene distribution, freshwater samples were collected from a drinking water treatment plant upstream of the North Han River where the 2-MIB odor was reported to occur in November, 2018 (Figure 1). The morphology of the vegetative cells in the water samples was characterized using phase-contrast microscopy (Axioskop2, Carl Zeiss, Jena, Germany). The average and standard deviation of the vegetative cell length and width were calculated using cell images taken under 1000-fold magnification. The cyanobacterial cells in the uni-cyanobacterial culture were separately collected using the micropipetting method [14] and cultivated in BG-11 medium at 26 °C under a 16:8 h light/dark cycle.

### 2.2. Monitoring 2-MIB Production in the Freshwater Samples

The reason for the odor issues in the North Han River were investigated by monitoring the odor-causing compounds in the freshwater samples taken during all seasons in 2018. Freshwater samples were collected from the influent source water in the drinking water treatment system near the river side (Figure 1). Then the odor-causing compounds, including geosmin and 2-MIB, were monitored in the freshwater samples using a GC-MS instrument.

The 2-MIB concentration in the culture medium was quantified using a GC/MS instrument (Agilent 5975C, Agilent Technologies, Santa Clara, CA, USA), according to the method published by the Korean Ministry of Environment regarding the measurement of drinking water monitoring items (25 June 2019). A Stir-barsorptive extraction system (SBSE system) (MPS2, Multi-Purpose Sampler, Gertel GmBH, Worms, Germany) was used to pretreat the liquor samples. Briefly, 15 mL of each sample was transferred into a 20 mL fresh SBSE vial and stirred at 1200 rpm for 120 min using a bar stirrer (length: 10 mm and thickness: 3.2 mm) for 2-MIB extraction. The stirrer bar was then dehydrated and placed in a thermal desorption system (TDS-2 system) at 280 °C for 3 min to detach the adsorbed components. The desorbed 2-MIB was concentrated at −120 °C using liquid nitrogen in the CIS PTV (Gerstel, Mülheim, Germany) of the GC injector and analyzed using the GC/MS system under the conditions described in Table 1.

### 2.3. DNA Extraction, PCR Amplification, and Phylogenetic Analysis

Cyanobacterial total DNA was extracted from both the cultured isolates and the water samples. The cyanobacterial biomass from the water samples was collected by filtering out 100 mL of the sampled water using a 0.22 μm pore size filter column which was then washed three times with sterilized water. Total DNA was extracted using a water DNA purification kit (Norgen, Thorold, ON, Canada).

For the phylogenetic analyses, PCR amplification was carried out using primer sets targeting the *mib*C gene and 16S rRNA with the 16S-23S rRNA gene ITS region (Table 2 and Figure S1). For the *mib*C gene, 1194 bp of the *mic* gene region was targeted in the 5133 bp part of the 2-MIB synthesis gene operon to generate the PCR primer set (Figure S1). The PCR amplicons were cloned using a TOPcloner PCR cloning kit (Enzynomics, Daejeon, South Korea) and sequenced at Macrogen (Macrogen, Seoul, South Korea). Phylogenetic trees were inferred using the maximum likelihood algorithm to assess the phylogenetic affiliation of the sequences (both 2-MIB and 16S rRNA with the 16S-23S rRNA gene ITS region) retrieved in this study. Phylogenetically similar sequences to the retrieved sequences (i.e., reference sequences) were collected from the GenBank database using NCBI BLAST [27,28] to construct a tree. The retrieved sequences and closest relative reference sequences were aligned using ClustalW [29], and the aligned sequences were used to construct a phylogenetic tree using MEGA X software [30] according to the manufacturer’s instructions. The relative evolutionary distances among sequences were calculated according to the Tamura 3-parameter model [31] and the Hasegawa-Kishino-Yano model [32] for each *mib*C and 16S rRNA with 16S-23S rRNA ITS genes, respectively. Tree topology was evaluated statistically using 1000 bootstrap resamplings.

### 2.4. Evaluation of 2-MIB Production during Cultivation of Isolates

Two experimental procedures were performed to investigate the 2-MIB production characteristics of the mat formed by the isolate under laboratory conditions. The first set investigated the properties of 2-MIB released from the cells of the formed mat. In this experiment, the isolate was inoculated in 25 mL of BG-11 medium using a 50 mL culture vessel. After the cyanobacterial mat had formed on the bottom wall of the culture vessel and 2-MIB production had begun, the culture medium was discarded, and the mat was washed three times with distilled water. Then, 25 mL of fresh BG-11 medium was added and incubated. These processes were repeated twice and the 2-MIB concentrations were measured during the process. The second procedure was used to investigate the characteristics of 2-MIB concentration change when the culture temperature decreased from 26 to 12 °C as the temperature changed from summer to autumn. The isolate was cultivated in 1.0 L of BG-11 medium in culture vessels (1.5 L), and the 2-MIB concentrations were monitored.

### 2.5. Whole Genome Sequencing

The whole genome extracted from the isolate was sequenced in a previous study by Jeong et al. [34]. The information was used to elucidate the genetic and biochemical backgrounds of 2-MIB production in the isolate. Briefly, the genomic DNA was extracted from the culture medium using a DNeasy PowerSoil kit (Qiagen, USA), and sequenced using the PacBio II platform and Illumina HiSeq 2500 (Macrogen, Korea). The draft sequence was assembled using FALCON-integrate v2.1.4 [35] and the genome was annotated using the NCBI Prokaryotic Genome Annotation Pipeline (PGAP) v.4.8 [36,37]. The gene sequences of the putative 2-MIB biosynthesis operon were compared with those in the NCBI database.

## 3. Results and Discussion

### 3.1. Characteristics of the Study Site

Paldang Lake, located in the mainstream of the North Han River, is one of the largest water sources in the metropolitan area of Seoul, South Korea, and is also one of the three main sources of inflow water (Figure 1). Odor issues caused by cyanobacteria sometimes occur in the largest drinking water sources. Previously, a large number of *Dolichospermum circinalis* (Rabenhorst ex Bornet & Flahault), a cyanobacterium that was recently re-classified and known to produce geosmin [38], occurred in the water system of Paldang Lake in the North Han River from November to December 2011 [39]. As a result, residents living in metropolitan areas suffered from an unpleasant odor in tap water. A strain of the *D. circinalis* species was isolated from the water samples with odor and considered as the causative organism at that time [40]. Moreover, from 2014 to 2017, the geosmin synthesis genes from two planktonic (*D. crassa, D*. *planctonica*) and one benthic (*Oscillatoria princeps*) cyanobacteria were identified in the source freshwater system. Finally, in November 2018, a high 2-MIB concentration was observed in the source water of the North Han River for the first time; however, only a limited distribution of cyanobacteria was reported.

### 3.2. Odor Identification and Quantification in Freshwater Samples

The monitoring results revealed that the odor-producing compounds were detected from June to December, 2018 in the freshwater samples of the North Han River. The concentrations of the compounds were below the detection limit from January to May in 2018. Interestingly, 2-MIB was the major compound detected during the summer and winter seasons, indicating that the odor issue in the river might be due to increased 2-MIB production.

**Figure 2:**
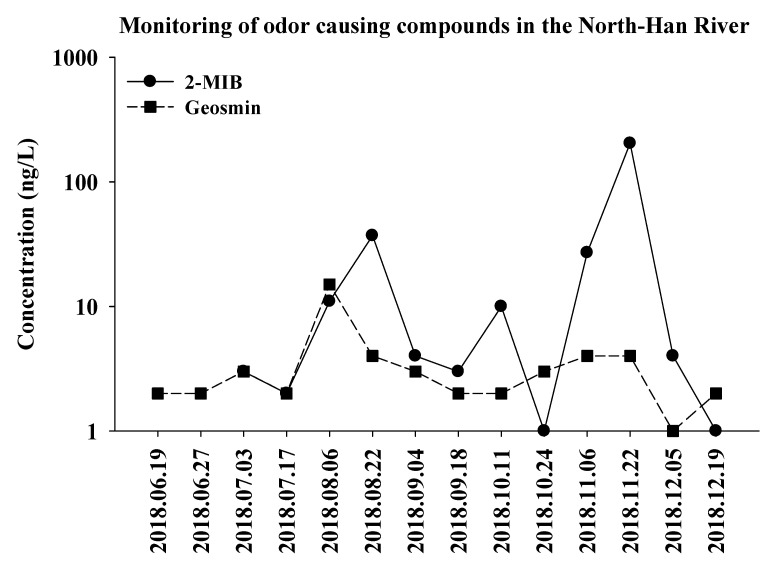
Monitoring of odor components in the freshwater samples of the North Han River, 2018. The concentration of each 2-MIB and Geosmin is represented with clear and gray area.

In the freshwater samples collected from the North Han River in 2018 (Figure 2), the 2-MIB concentration showed gradually increased patterns from June, with the highest concentration (up to 204 ng/L) detected in the samples collected from November, when the temperatures were low (ranging from 10.3 to 13.7 °C). These results suggest that the earthy-musty odor issue in the North Han River could mainly be caused by microbes that produce 2-MIB compounds during the summer season, which are then increasingly released during low temperature conditions.

### 3.3. Distribution of 2-MIB Synthesis Gene in Odor Emitting Samples

**Figure 3:**
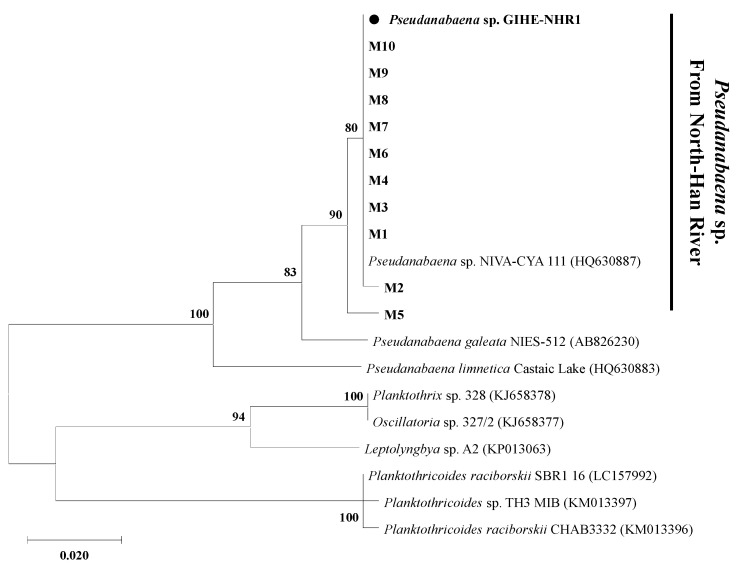
Maximum likelihood tree based on *mib*C gene sequences. The evolutionary history was calculated using the Maximum Likelihood method and the Tamura 3-parameter model. A discrete gamma distribution was used to model evolutionary rate differences among sites. M1~M10 are *mib*C gene sequences obtained by cloning from the North Han River samples.

After PCR amplification, positive *mib*C gene amplicons were observed in the template DNA extracted from the freshwater sample (data not shown). The amplicons were purified and used to construct the gene clone library so that the results could be analyzed in detail. Phylogenetic analyses of sequenced *mib*C genes showed that all of the observed 2-MIB gene sequences showed 100% similarity with the reference gene retrieved from the single microorganisms (Figure 3). These results indicate that the 2-MIB odor in the North Han River freshwater samples in 2018 might be derived from a single producer microorganism. In addition, the NCBI BLAST search results revealed that the *mib*C gene sequences identified in this study showed 100% similarity with those observed in *P. yagii* (HQ630887) isolated from Lake Biwa, Japan (Figure 3). However, the phylogenetic analyses of the *mib*C gene sequences revealed that the *mib*C genes observed in this study clustered together, but showed separated deep branches compared to those retrieved from other *Pseudanabaena* species (Figure 3). This suggested that candidate 2-MIB producing *Pseudanabaena* species might be the only 2-MIB odor producer in the North Han River freshwater samples taken in this study.

### 3.4. Isolation and Characterization of 2-MIB Pseudanabaena Species

**Figure 4:**
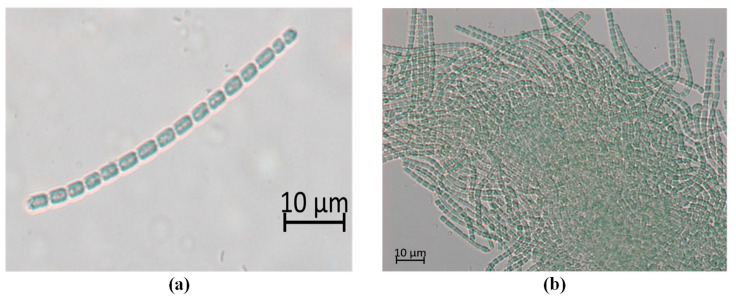
Phase contrast optical micrograph of *Pseudanabaena yagii* GIHE-NHR1 from the North Han River showing (**a**) its natural state, and (**b**) the mat form.

The 2-MIB producing *Pseudanabaena* species from the 2-MIB odor-issue freshwater samples were isolated by separating out the benthic cyanobacteria that were considered to be morphologically affiliated with *Pseudanabaena*-like species from the rest of the sample (Figure 4). Interestingly, under laboratory culture conditions, the isolated *Pseudanabaena*-like species could grow as solitary trichomes; however, most of the members of this species grow as mat forms. The color of the isolated trichomes was blue-green; the shape of the vegetative cells was straight or slightly curved; and the cross-wall had constrictions. In contrast, the cells did not show a sheath shape. The length and width of the trichomes were 4.8 ± 1.4 μm and 1.5 ± 0.1 μm, respectively, and the width was almost constant. An aerotope was also observed on both sides of the vegetative cells.

Generally, *Pseudanabaena* cells are non-motile, but sometimes show trembling movement in microscopy observations. According to a previous report by Jeong et al. [33], the nifH genes for nitrogen fixation are included in the genome. However, a heterocyst for nitrogen fixation was not observed at the end of the trichome isolated in this study. This morphological feature was consistent with *P. yagii* that was isolated from Lake Biwa in Japan and is known to produce 2-MIB according to the molecular sequencing results [41]. Furthermore, previous studies reported that although 2-MIB could also be produced by non-*Pseudanabaena* species [42,43,44], *Pseudanabaena*-like species are known to be one of the major cyanobacteria that can produce 2-MIB from their non-heterocystous filamentous structures [11,45]. These results suggest that molecular phylogenetic analyses should be used to clearly identify the taxonomic class of the isolated species before further analyses.

Previously, morphological characteristics, and 16S rRNA gene, 16S-23S rRNA ITS sequences, and secondary structure analyses have been used to taxonomically classify *Pseudanabaena* species [46]. Based on these identification methods, 45 *Pseudanabaena* species were taxonomically identified and reported in the AlgaeBase database. To date, eight *Pseudanabaena* species have been reported to be present in the freshwater environment of South Korea, including *P. amphigranulata*, *P. catenata*, *P. galeata*, *P. limnetica*, *P. lonchoides*, *P. minima*, *P. mucicola*, and *P. westiana*. However, only *Pseudanabaena* sp., *P. cinerea*, *P. foetida*, *P. galeata*, *P. limnetica*, and *P. yagii* are known to produce the 2-MIB odor in freshwater environments [11,41,47,48,49].

**Figure 5:**
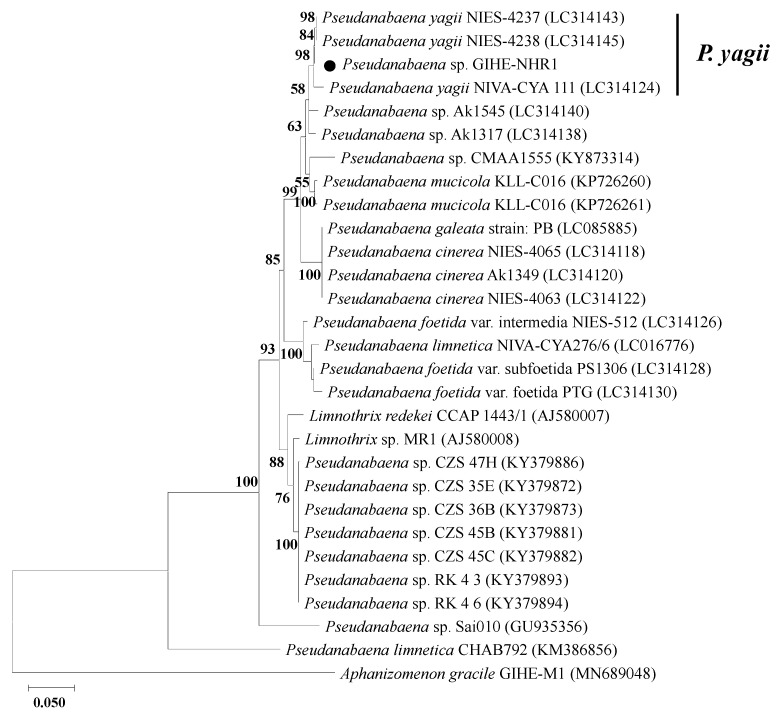
Maximum likelihood tree based on 16S rRNA + 16S-23S rRNA ITS sequences. The evolutionary history was inferred by using the Maximum Likelihood method and the General Time Reversible (GTR) model. The percentage of trees in which the associated taxa clustered together is shown next to the branches. Initial tree(s) for the heuristic search were automatically obtained by applying the Neighbor-Join and BioNJ algorithms to a matrix of pairwise distances estimated using the Maximum Composite Likelihood (MCL) approach. Then topology was selected and the superior log likelihood value. A discrete gamma distribution was used to model evolutionary rate differences among sites. Closed circle indicated the 16S rRNA + 16S-23S rRNA ITS sequences retrieved from the isolated species of this study.

Molecular phylogenetic analyses using the 16S rRNA gene and 16S-23S rRNA ITS sequences were performed to identify the taxonomic affiliation of isolated *Pseudanabaena*-like species (Figure 5). The phylogenetic analysis compared the 16S rRNA and 16S-23S rRNA ITS sequences of the isolated species with their closest relatives. The sequence retrieved from the isolate in this study was taxonomically affiliated to the *Pseudanabaena* genus and showed a deep branch with the closest relative, *P. yagii* (Figure 5). Based on the results of the phylogenetic analysis, the isolated species was named *Pseudanabaena yagii* GIHE-NHR1.

According to the morphological and molecular genetic characteristics, the isolated *P. yagii* that caused the 2-MIB odor in the North Han River has not been reported in the water environments of South Korea. Byun et al. [18] reported a significant statistical relationship between *P. limnetica* cell number and 2-MIB concentration in the North Han River. However, *P. limnetica*, which is a planktonic species, is morphologically distinct from the *P. yagii* GIHE-NHR1 isolated in this study, which showed benthic growth behavior. Furthermore, in their study, 2-MIB synthesis genes were not identified, and the ability to generate 2-MIB has not been directly characterized by cultivation-based studies. In contrast, Jeong et al. [33] reported that the isolate *P. yagii* GIHE-NHR1 included a 2-MIB biosynthesis gene cascade in its genome, suggesting that the *P. yagii* species might have been the major 2-MIB odor producer in the North Han River in 2018.

### 3.5. Characterization of P. yagii GIHE-NHR1 2-MIB Biosynthesis Genes

The MIB synthase sequence has been used to design primers to detect potential 2-MIB producing microorganisms [25,26] (Figure S1). A BLAST search of MIB synthase showed that the highest homology (99.74%) was found in *P. yagii* NIVA-CYA 111 (HQ630887) isolated in Japan. Among the MIB synthesis genes, two categories of enzymes: (1) geranyl diphosphate 2-methyltransferase (GPPMT) and (2) MIB synthase in an MIB operon, were reported to be mainly involved in the biosynthesis of 2-MIB in cyanobacteria [6]. According to a genome study of *P. yagii* GIHE-NHR1 by Jeong et al. [34], the draft genome sequence contains two major 2-MIB biosynthesis genes, which are located between two homologous cyclic nucleotide binding protein (*cnb*A and B) genes (Figure S1). Furthermore, the BLAST search results revealed that the 2-MIB synthesis genes observed in this study showed 95.79% sequence similarity with those observed in the *Pseudanabaena* sp. dph15 (Accession number: HQ830028) isolated from China. Wang et al. [50] reported that although it has not been completely confirmed yet, the *cnb* gene might be involved in the regulation of 2-MIB biosynthesis in cyanobacterial species, suggesting that the odor issue in the North Han River 2018 might be caused by the production of 2-MIB producing *Pseudanabaena* species.

To better understand the genetic characteristics of the 2-MIB synthesis genes, the amino acid sequences were compared with those observed in other cyanobacterial genomes (Table 3). Generally, it has been reported that 2-MIB biosynthesis proteins include two highly conserved magnesium binding motifs that are vital elements of all terpene cyclases but with a number of differences [51]. Wang et al. [50] reported that 2-MIB synthase from other microorganisms, which are typical Mg^2+^-binding motifs, showed the **DD**xxx**E (or D)** and **N**Dxx**S**xxx**E** sequences. The protein sequences of the last two amino acids in motif1 were divided into three groups (TE, SE, and SD) (Table 3). Interestingly, two motifs of MIB synthase observed in the *P. yagii* GIHE-NHR1 isolate showed the sequence **DD**YYA**D**DTE and **N**DLL**S**VAK**D**. Among the six *Pseudanabaena* species, the 2-MIB synthase protein sequences in motif 1 showed similar sequence orders, except for strains dqh15 and *P. limnetica* str. Castaic Lake and *P. galeata* NRERC-312, whereas those observed in other cyanobacterial species showed variable regions at the end of motif 1. In addition, in the case of the genus Planktothricoides, the last **E** (glutamic acid) was replaced by a **D** (aspartic acid), although the other protein sequences for this motif were consistent with the typical sequences for MIB synthase.

Compared to motif 1, the protein sequences in motif 2 were divided into two types across the cyanobacterial species. In this motif, variations in the protein sequences were only observed at the third position from the end. In the case of the *Planktothricoides*, *Microcoleus*, and *Oscillatoria* genera, the A (alanine) sequence at the last third position was replaced by N (asparagine). Zhou and Peters [52] suggested that although it has not been confirmed in the case of cyanobacterial species, these variations could affect the catalytic activity of diterpene cyclase in plants. The results from this study suggest that the 2-MIB synthesis genes included in most of the cyanobacterial species might have similar structural and functional characteristics, and the odor issue in the North Han River might be caused by the 2-MIB producing cyanobacterial species.

### 3.6. Characteristics of 2-MIB Production in P. yagii GIHE-NHR1

**Figure 6:**
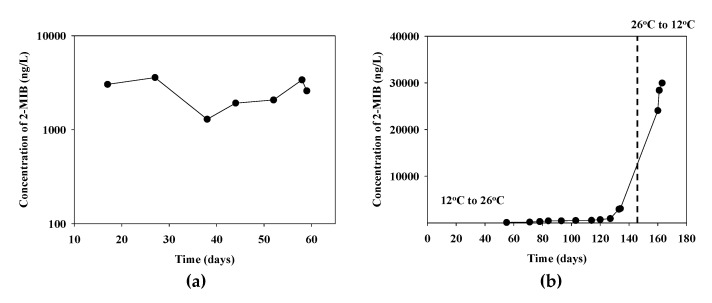
Changes of 2-MIB concentration (**a**) in the fresh culture media from the cyanobacterial mat wash and (**b**) in the mat formed cyanobacterial cultures under the temperature variation conditions.

The 2-MIB production characteristics of the *P. yagii* strain GIHE-NHR1 isolate were assessed by monitoring the 2-MIB production patterns in response to temperature changes using a GC-MS system (Figure 6). The expression patterns of the *mib*C genes in these biomass samples were quantified (Figure S2). The mats were grown by inoculating the medium with *P. yagii* isolate and incubating them for 88 days. The 2-MIB concentrations were then measured.

The 2-MIB concentration reached approximately 3000 ng/L during the mat-forming growth periods, (Figure 6a). At that time, the medium was discarded, the mat was sufficiently washed to remove the remaining 2-MIB, and fresh medium was added. The 2-MIB concentration recovered to 3000 ng/L after 17 days of incubation, and reached 3600 ng/L and after 27 days of incubation. In the second round of incubation, the recovery rate was somewhat slow at the beginning; however, it reached 3400 ng/L a month later, which was similar to the first round (Figure 6a).

To investigate why high concentrations of 2-MIB occurred in the freshwater system during the autumn season when the temperature was low, the incubation temperature was changed from 12 to 26 °C during the incubation period and the 2-MIB concentration in the media was measured. The 2-MIB concentration varied from 120 ng/L (55 days) to 3100 ng/L (134 days). On the 154th day, the culture temperature decreased from 26 to 12 °C. The color of the mat changed from green to yellowish after lowering the culture temperature and this color change may indicate that the cyanobacteria are aging or dying. After 6 days of incubation at the lower temperature, the 2-MIB concentration increased to approximately 24,000 ng/L and to almost 30,000 ng/L on the 9th day (Figure 6b).

Gao et al. [53] reported that the growth of *Pseudanabaena* sp. was closely related to the temperature, pH, chemical oxygen demand, and NH_3_-N concentration of the water environment. The optimum temperature for the growth of *Pseudanabaena* species ranged from 20 to 30 °C, which was observed in the freshwater environment during spring and summer. In addition, Wert et al. [54] reported that the accumulated, cell-bound 2-MIB could be released from disintegrated cells by a combination of grazing and environmental stresses, such as desiccation and oxidation. These results suggest that 2-MIB producing *Pseudanabaena* species might be sensitive to water temperature.

Interestingly, a high *mib*C gene expression level was observed under all experimental conditions. In particular, regardless of the temperature decrease, *mib*C gene expression changed in a limited manner even when the incubation temperature decreased at low temperatures (Figure S2). Kakimoto et al. [48], who studied 2-MIB production and the expression of relative genes in response to temperature changes in *P. galeata*, reported that the expression of both the 2-MIB synthesis gene and the intracellular concentration of 2-MIB increased at 30 °C compared to 20 °C. These results suggest that the rapidly increasing pattern for 2-MIB concentration during low-temperature incubation might be due to the release of cell-bound 2-MIB into the water from broken and dead cells.

Taken together, these results suggest that in the North Han River during November, 2018, as the water temperature decreased, benthic cyanobacteria in the mat died and the cells disintegrated, which released all the cell-bound 2-MIB at once. For these reasons, the concentration of 2-MIB in water might increase rapidly and cause odor problems, including in the tap water distribution system.

## 4. Conclusions

In this study, *P. yagii*, which is known to be a 2-MIB odor-causing cyanobacteria, was first isolated from the freshwater of the North Han River, South Korea, by polyphasic analysis. The results produced after measuring the odor-causing compounds in freshwater samples revealed that the muddy odor issue in the North Han River might be mainly caused by microbes that produce 2-MIB compounds during the summer season and release them under low temperature conditions. Only one type of 2-MIB synthesis gene (*mib*C) was detected in the river water samples and was 100% identical to that of *P. yagii* isolated from Biwa Lake in Japan. Candidate 2-MIB producing benthic cyanobacteria were isolated from the 2-MIB odor-issue freshwater samples using a microscopic method. Phylogenetic analysis of the 16S rRNA + 16S-23S rRNA ITS region of the isolated species revealed more than 99% homology with *P. yagii* and showed a deep branch in the phylogenetic tree. The results for 2-MIB production after cultivation of the *P. yagii* GIHE-NER1 isolate also revealed that the 2-MIB concentration increased when the *Pseudanabaena* species began mat growth, and showed a rapidly increasing pattern during incubation at a low temperature, which suggested that the isolated, 2-MIB producing *P. yagii* GIHE-NER1 might form a mat at the bottom of the river during the summer season and then release a certain amount of 2-MIB into the water. Furthermore, when the water temperature decreased, the dead cells from the mat rapidly released large amounts of cell-bound 2-MIB into the water. In summary, the physiological and molecular genetic characteristics of 2-MIB production by *P. yagii* GIHE-NER1 were investigated for the first time in this study.

## Figures and Tables

**Table 1:** Analytical conditions for detecting 2-MIB concentrations using GC/MS.

GC (7890A, Agilent)	MS (5975C, Agilent)
Column: HP-5MS	SIM Mode
30 mm (L) × 0.25 mm (ID) × 0.25 μm	Selected ion
	2-MIB (95,108,135)
**Oven Temp.**	
Initial Temp. 40 °C, Hold 5 min	
1st rate 20 to 65 °C/min (5 min)	
2nd rate 8 to 215 °C/min	
3rd rate 30 to 300 °C/min (3 min)	

**Table 2:** PCR primer sets used in this study.

Name	Target	Sequence (5′ to 3′)	Product Size (bp)	Annealing Temp. (°C)	Reference
MIBS02F	Monoterpene cyclase *mib*C	ACCTGTTACGCCACCTTCT	307	63	[26]
MIBS02R		CCGCAATCTGTAGCACCATG			
16S27F	16S rRNA + 16S-23S r/RNA ITS	AGAGTTTGATCCTGGCTCAG	2080	57	[33]
23S30R		CTTCGCCTCTGTGTGCCTAGGT			

**Table 3:** Comparison of protein sequence motifs for 2-MIB biosynthesis in 2-MIB producing microbes.

Cyanobacterial Strain	Motif 1	Motif 2
*Pseudanabaena yagii* GIHE-NHR1	-DDYYADD**TE-**	-NDLLSV**A**KD-
*Pseudanabaena yagii* NIVA-CYA 111	-DDYYADD**TE-**	-NDLLSV**A**KD-
*Pseudanabaena galeata* NIES-512	-DDYYADD**TE-**	-NDLLSV**A**KD-
*Pseudanabaena* sp. dqh15	-D**G**YYADD**TE-**	-NDLLSV**A**KD-
*Pseudanabaena limnetica* str. Castaic Lake	-DDYYADD**SE-**	-NDLLSV**A**KD-
*Pseudanabaena galeata* NRERC-312	-DDYYADD**SE-**	-NDLLSV**A**KD-
*Planktothricoides raciborskii* CHAB3331	-DDYYADD**SD-**	-NDLLSV**N**KD-
*Planktothricoides raciborskii* BWN4	-DDYYADD**SD-**	-NDLLSV**N**KD-
*Microcoleus pseudautumnalis* Ak1609	-DDYYADD**SE-**	-NDLLSV**N**KD-
*Oscillatoria limosa* LBD	-DDYYADD**SE-**	-NDLLSV**N**KD-
*Leptolyngbya* sp. A2	-DDYYADD**SE-**	-NDLLSV**A**KD-
*Oscillatoria* sp. 327/2	-DDYYADD**SE-**	-NDLLSV**A**KD-
*Planktothrix* sp. 328	-DDYYADD**SE-**	-NDLLSV**A**KD-
*Oscillatoria prolifera* OpA	-DDYYADD**SE-**	-NDLLSV**A**KD-

## Data Availability

Not applicable.

## Supplementary Materials

The following are available online at https://www.mdpi.com/article/10.3390/microorganisms9122486/s1, Figure S1: 2-MIB synthesis operon and target region for PCR detection, Figure S2: Quantification of 2-MIB producing gene expression during temperature variation conditions using qPCR.
