# Supplementary material for: 2-Methylisoborneol (2-MIB) Excretion by Pseudanabaena yagii under Low Temperature
Source: Microorganisms. 2021 Nov 30;9(12):2486. doi: 10.3390/microorganisms9122486 (PMC8705757; doi:10.3390/microorganisms9122486)
Supplement: Supplementary file 1 [file microorganisms-09-02486-s001.zip › microorganisms-1386119-supplementary.pdf]

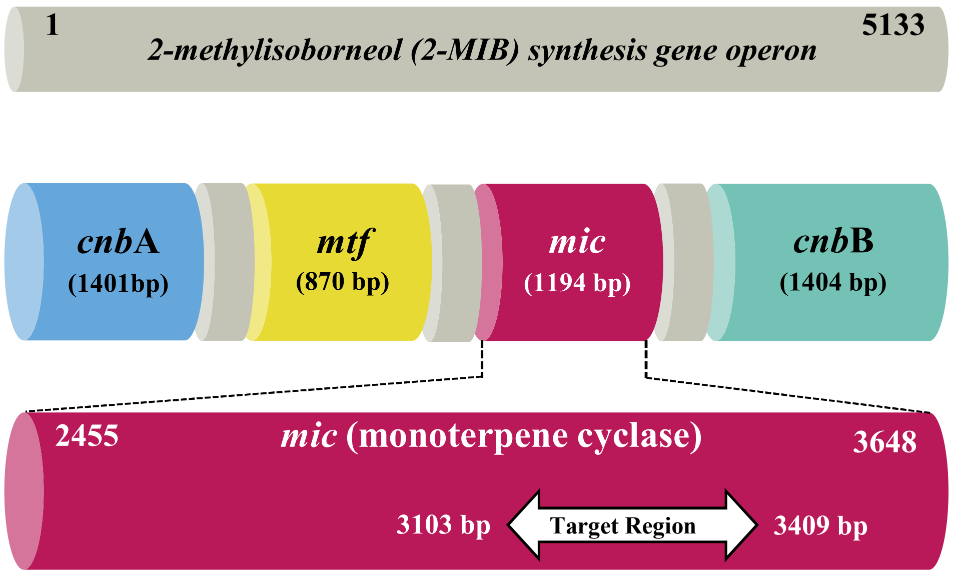

Figure S1. 2-MIB synthesis operon and target region for PCR detection.

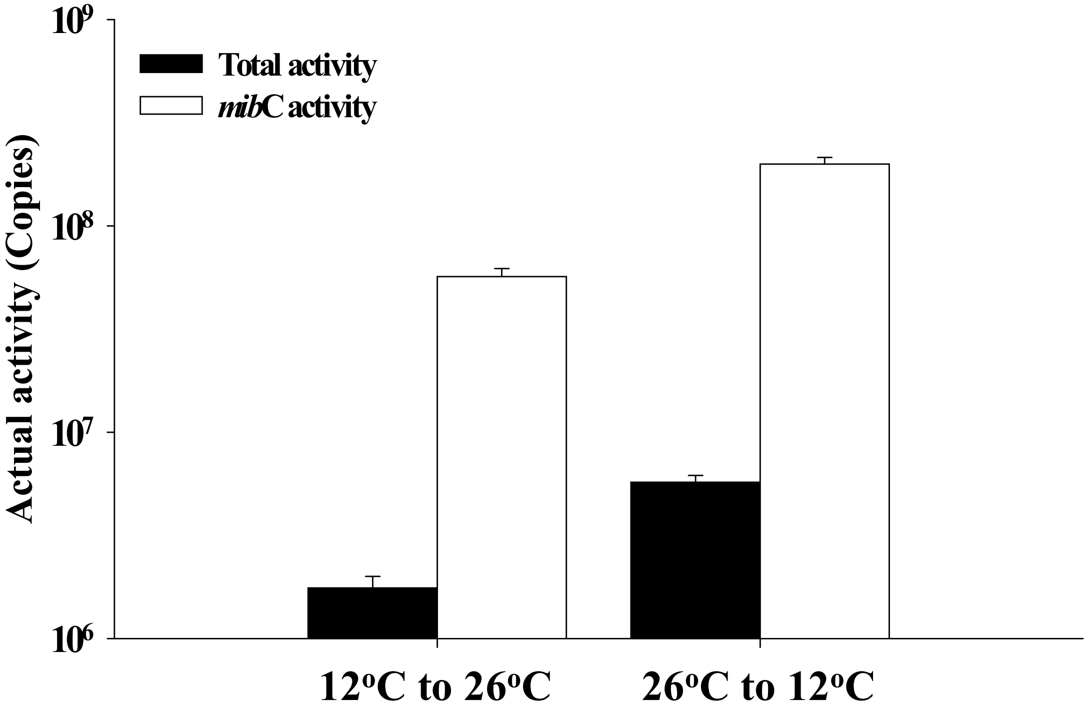

Figure S2. Quantification of 2-MIB producing gene expression during cell growth under temperature variation conditions.
